# A Case of Acute Life-Threatening Pulmonary Hemorrhage from Synthetic Cannabinoid Abuse

**DOI:** 10.1155/2019/8137648

**Published:** 2019-05-20

**Authors:** Muhammad Imtiaz, Biplab Saha, Uzma Sana Ullah, Aditi Saha

**Affiliations:** ^1^Division of Pulmonary and Critical Care Medicine, Conemaugh Memorial Medical Center, Johnstown, Pennsylvania, USA; ^2^Division of Pulmonary and Critical Care Medicine, Albany Medical Center, Albany, New York, USA; ^3^Department of Medicine, Conemaugh Memorial Medical Center, Johnstown, Pennsylvania, USA; ^4^Department of Medicine, Saint Barnabas Medical Center, Livingston, New Jersey, USA

## Abstract

We report a case of alveolar hemorrhage secondary to inhalation of synthetic cannabinoid. The patient developed hemoptysis and respiratory failure 48 hours after the episode. Alveolar hemorrhage from synthetic cannabinoid use is a rare entity that has been reported only thrice previously. The unique feature of this case was that the initial urine and blood toxicology screens were negative for cannabinoids and the diagnosis was confirmed via detection of serum metabolites of a synthetic cannabinoid.

## 1. Background

Use of synthetic cannabinoid (SC) is on the rise. Numerous new compounds with minimal chemical modification and similar biologic activity have been designed over the years. Many of these SC compounds are now considered schedule 1 controlled substances. Most of the SC abused in the United States are produced overseas and are often available to the consumers over the internet or sold as “fake marijuana” at head shops. A prompt diagnosis of SC toxicity requires high index of suspicion in the right clinical setting as well as an appropriate work-up. Diffuse alveolar hemorrhage (DAH) is a life-threatening condition that predominantly stems from infectious, inflammatory, coagulopathy, and cardiac etiology [1]. DAH secondary to inhalational toxin exposure has also been reported [2, 3]. We report a case of DAH after SC use in a 27-year-old man. A unique aspect of this case was that a urine drug screen for tetrahydrocannabinol (THC) was negative and the diagnosis was confirmed by the presence of the serum metabolite of a SC, UR-144.

## 2. Case Presentation

A 27-year-old man with history of polysubstance abuse was witnessed to inhale “K2,” a synthetic cannabinoid. Over the next hour, he became unresponsive and was brought to an emergency room where he was found to be hypoxemic. There was no evidence of traumatic injury. He was intubated and admitted to the intensive care unit (ICU).

He had no other significant past medical history. Physical examination revealed an intubated and sedated patient; temperature was 97.1 °F, blood pressure was 144/84 mmHg, pulse was 98 beats/min, and oxygen saturation was 100% on FiO_2_ 0.5; and bilateral coarse crackles were audible on chest auscultation.

**Figure 1:**
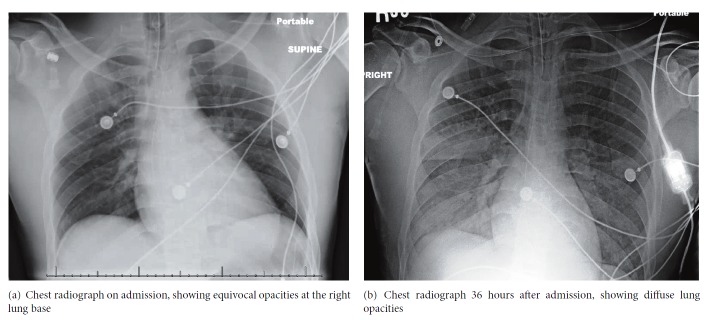


Laboratory evaluation revealed WBC 10,900/dL, hemoglobin 12.6 g/dL, hematocrit 39.8 %, platelets 191,000/dL, sodium 140 meq/L, potassium 3.7 meq/L, chloride 102 meq/L, bicarbonate 19 mmol/L, BUN 13 mg/dL, creatinine 1.2 mg/dL, and creatine kinase 1,952 IU/L. Computed tomography (CT) of the brain showed no acute intracranial pathology. His initial arterial blood gas (ABG) values were pH 7.28, pCO_2_ 58 mmHg, and pO_2_ 125 mmHg on 50% oxygen. Chest radiography revealed alveolar opacities in the right upper lobe (Figure 1(a)).

**Figure 2:**
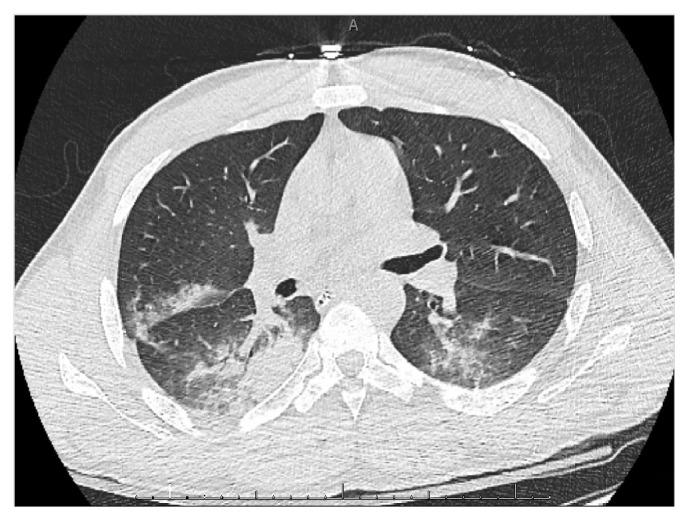
Computerized tomography (CT) of the chest 36 hours after admission, showing bilateral parenchymal opacities.

The patient was started on broad-spectrum antibiotics. A urine and blood toxicology screen was positive for benzodiazepines (which he received after intubation) and negative for amphetamines, barbiturates, cocaine, opiates, phencyclidine, methadone, and cannabinoids. On the second day, frank blood was noted on suction from the endotracheal tube. His gas exchange worsened requiring a FiO_2_ 1.0 to maintain adequate oxygenation. A blood gas showed profound hypoxemia with a pO_2_ 110 mmHg. A chest radiograph revealed worsening bilateral alveolar infiltrates (Figure 1(b)). A CT of the chest revealed patchy ground glass opacities and diffuse lung consolidation (Figure 2). Bronchoscopy was performed and showed oozing of blood from all right lung airways and the left lower lobe bronchus. Sequential bronchoalveolar lavage (BAL) confirmed diffuse alveolar hemorrhage by demonstrating increasingly bloody return. Hemosiderin laden macrophages were seen in BAL fluid on microscopy. Measurements of serum anti-nuclear antibody, anti-neutrophil cytoplasmic antibody, and anti-glomerular basement membrane antibody were negative. Urine analysis was negative for hematuria. BAL was negative for an infectious etiology. An echocardiogram was normal. There was no evidence of coagulopathy. UR- 144 N (4/5-hydroxypentyl), a metabolite of UR-144, was identified in the patient's blood by qualitative enzyme-linked immunosorbent assay (ELISA).

The patient was empirically treated with high-dose steroid for 3 days, followed by prednisone 40 mg/day that was tapered to 10 mg/day over 4 days and then discontinued. Over the 48 hours after corticosteroid administration, his oxygenation improved significantly. A chest radiograph performed 96 hours after admission showed complete resolution of the alveolar opacities. The patient was successfully extubated and transferred out of ICU. He was discharged 10 days after admission, neurologically and functionally intact.

## 3. Discussion

We have presented a patient with alveolar hemorrhage from inhalation of UR-144, a SC. SC abuse is becoming more frequent [4]. Most users are young and have a history of marijuana or polysubstance abuse [5]. There are very few reports documenting potential life-threatening respiratory conditions developing after SC inhalation, including diffuse alveolar hemorrhage and respiratory failure in otherwise healthy individuals. In previous cases [4, 6, 7] as well as ours, no other identifiable etiology for alveolar hemorrhage was found, suggesting that SC was the cause. In all these cases, alveolar hemorrhage developed within 24-48 hours after SC inhalation, suggesting a temporal relationship.

Synthetic cannabinoids interact with CB_1_ and CB_2_ receptors and are more potent than their natural counterpart, tetrahydrocannabinol (THC) [8]. The exact mechanism by which these compounds cause alveolar hemorrhage is currently unknown. The most likely explanation is direct injury to the alveolocapillary membrane following inhalation. What was uniquely important in our patient's case was that the initial urine drug screen for cannabinoids was negative, and yet a serum metabolite of UR-144, UR-144 N (4/5-hydroxypentyl), was identified. Therefore, a negative urine drug screen for THC does not exclude the possibility of SC toxicity and a dedicated urine or serum study is necessary [8, 9].

We believe that the diagnosis of synthetic cannabinoid-induced respiratory failure can be established by the acute onset of hemoptysis and respiratory failure that is delayed 24-48 hours after inhalation of the drug, provided that alternative conditions including infection, coagulopathy, malignancy, autoimmune, or connective tissue diseases have been excluded. The presence of UR-144 metabolite in serum solidifies the diagnosis, although the diagnosis can be usually made on clinical grounds. Treatment remains supportive. The use of corticosteroids may be of benefit, although presently this remains conjectural.

## 4. Conclusion

DAH from synthetic cannabinoid use is rare. Clinicians need to be aware of the time delay between synthetic cannabinoid inhalation and the onset of respiratory failure. A negative cannabinoid urine screen does not exclude the diagnosis and a serum and urine metabolite screening is necessary to identify the culprit compound.
